# Performance Streaks in Elite Beach Volleyball - Does Failure in One Sideout Affect Attacking in the Next?

**DOI:** 10.3389/fpsyg.2019.00919

**Published:** 2019-04-30

**Authors:** Daniel Link, Sebastian Wenninger

**Affiliations:** Department of Exercise Science and Sports Informatics, Technical University Munich, Munich, Germany

**Keywords:** performance analysis, notational analysis, psychological momentum, performance streaks, action sequences

## Abstract

This study explores the influence of sideout failure on performance in the next sideout in beach volleyball. The sample comprises 965 elite matches in the FIVB World Series 2012–2016 and in the Olympic Games 2012/2016 including 28,974 sideout sequences (12,755 for men and 16,219 for women). A sideout sequence consists of two sideouts by the same player during the same set in a timeframe of four rallies. The first sideout in this sequence is referred to as the previous sideout and the second sideout as the next sideout. After misses, $\chi^{2}$-tests indicate a significantly higher technique alternation rate (from spike to shot or vice versa) in the next sideouts for both men (+32.7%) and women (+40.4%) than the next sideouts after hits. After shot misses, the share of shots in the next sideouts was −12.9% lower for men and −8.3% lower for women than the next sideouts after shot hits. After spike misses, the share of shots in the next sideouts by female players was +5.5% significantly higher, and shot hit rate was −6.5% lower than the next sideouts after spike hits. These findings support the belief that tactical decisions and performance in top-level beach volleyball are influenced by failure in the previous sideouts. They might support coaches and players when analyzing matches and developing game strategies.

## Introduction

Beach volleyball is a clearly structured game with two basic situations. In a typical *sideout*, one team receives the opponent’s serve, passes the ball, and tries to score with an attack (Koch and Tilp, 2009a). The attacking player can usually choose between a hard-driving *spike* and a soft *shot*, which is preferably played over the block to the open part of the court. The serving team is in *defense* and tries to defend the attack by blocking/digging the attack and to score itself. The winner of a rally will be the next to serve, so that sideout and defense switch continuously between the teams (FIVB, 2017). While the serving player must be alternated every time this occurs, the player that receives the serve can be the same in each sideout of a team.

Recent years have seen many studies on beach volleyball covering different aspects of the games. Research has dealt with the efficiency of game actions and techniques with regard to age, gender, and player’s role (Jimenez-Olmedo et al., 2012; Medeiros et al., 2014; Palao and Ortega, 2015; Šimac et al., 2017). Other studies have focused on perception and anticipation (Cañal-Bruland et al., 2011; Güldenpenning et al., 2013; Klostermann et al., 2015; Noël et al., 2016) and the influence of rule changes (Giatsis, 2003; Ronglan and Grydeland, 2006; Palao et al., 2012). Research has also examined physical workload (Palao et al., 2014; Hank et al., 2016) and the biomechanical aspects of beach volleyball (Tilp et al., 2008; Giatsis et al., 2018).

One question, which has not been investigated so far, is whether failure in a sideout affects the behavior of players in the next sideout. In elite beach volleyball, a team is more likely to score from a sideout situation than from a defense situation (Koch and Tilp, 2009b; Giatsis et al., 2015; Sicoli et al., 2016). The reason is that reception of a serve is much easier and more controllable than defending an attack (Costa et al., 2011) because the ball is hit closer to the net and reaction time is less. A rally won by the serving team is therefore called a “break,” which is in contradistinction to many other sports (e.g., tennis). When a player does not score from sideout, there is an increased need and may be a psychological pressure to convert the next sideout. Since there are only two players per team in beach volleyball and substitutions are not allowed, the responsibility of each player is higher than other team sports such as volleyball. For this reason, many athletes and coaches believe that players make different tactical decisions after sideout failures when compared to successful ones.

Related questions have already been addressed for other sports under the headline of “hot-hand” research. The hot hand in sports refers to the belief that success breeds success in a sequence of actions. Gilovich et al. (1985) were the first to ask in relation to basketball if the probability of a hit after a sequence of successful shots is higher than after misses. Subsequently numerous studies have tried to find evidence for the existence of the hot-hand phenomena in various sports, including archery (Medeiros Filho et al., 2008), baseball (Vergin, 2000), bowling (Yaari and David, 2012), cycling (Perreault et al., 1998), dart (Stins et al., 2018), golf (Clark, 2003), tennis (Jackson and Mosurski, 1997), and volleyball (Raab et al., 2012).

Results provide an inhomogeneous picture. One group of studies finds evidence against the existence of hot hand (e.g., Gilovich et al., 1985; Vergin, 2000; Bocskocsky et al., 2014; Csapo and Raab, 2014), whereas a second group maintains the contrary (e.g., Raab et al., 2012; Bocskocsky et al., 2014; Miller and Sanjurjo, 2014; Stins et al., 2018). Meta-studies provide comprehensive overviews of results and associated methodological issues and mostly agree that evidence for the hot-hand phenomena is controversial and fairly limited (Bar-Eli et al., 2006; Avugos et al., 2013; Iso-Ahola and Dotson, 2014). To our knowledge, only one previous study has focused on negative streaks, the so-called “cold hand” (Köppen and Raab, 2012). Here, the authors reported that volleyball playmakers passed fewer balls to a player who performed a sequence of misses. For beach volleyball, no studies on the hot hand or cold hand exist. There are some studies dealing with the sequence effects in a broader sense – influence of rally length and reception quality on attacking performance (Koch and Tilp, 2009a; Sánchez-Moreno et al., 2015) – but these do not examine failure and dependencies between rallies.

This study examines whether failure in a sideout attack affects attacking performance in the next sideout by the same player. Three related subsidiary questions are asked: (1) does sideout failure affect probability of changing attacking technique, (2) does sideout attacking failure increase probability of a further attacking failure (“cold hand”), and (3) are these effects influenced by the attacking technique used? Answering these questions promises benefits for coaches and players, since this could underpin psychological interventions. In addition, many beach top-level volleyball teams perform match analysis in order to uncover typical patterns in opponents’ tactical behavior (Link, 2014). Scientific evidence for the general existence of sequence effects would provide an argument for looking for such streaks when developing match strategies. Therefore, the discussion also includes a single case example of how this can be conducted in practice.

## Materials and Methods

### Sample

In line with the objectives of the study, a non-participative observational approach was applied. The sample comprises 965 matches (413 men and 552 women) in the FIVB World Series 2012–2016 and the 2012 and 2016 Olympic Games. Since each player agreed to the video recording of matches on signing their player license, an ethics approval was not required as per applicable institutional and national guidelines. Nevertheless, all procedures performed in the study were in strict accordance with the Declaration of Helsinki as well as with the ethical standards of the local ethics committee.

### Performance Variables

**Figure 1:**
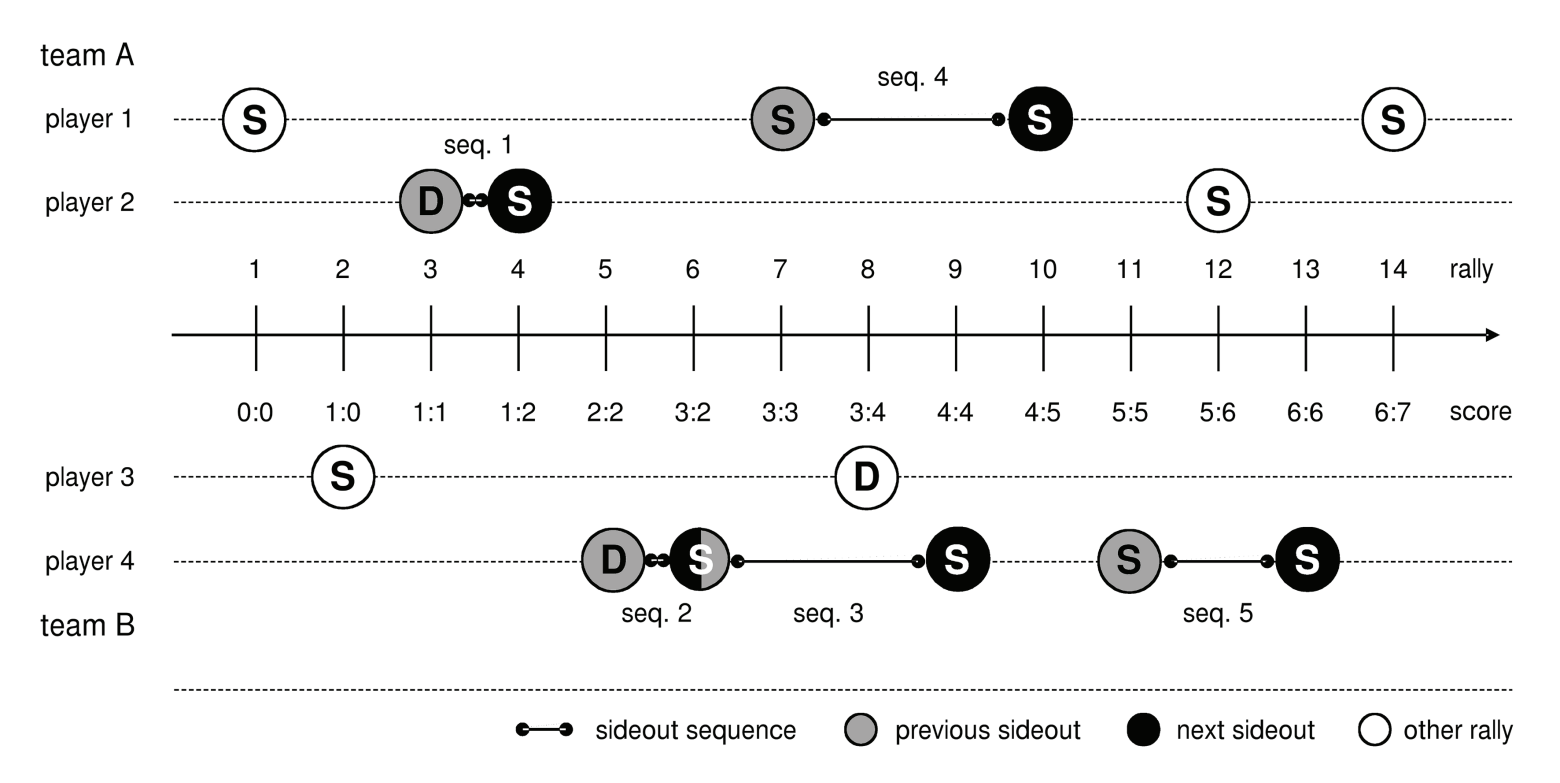
Concept of sideout sequences. Example showing all five sideout sequences (seq.) during the first 14 rallies (circles) in a match. Capital letters indicate whether the sideout team (S) or the serving/defending (D) team scored. In rally 3, the sideout is played by player 2, the serving team scored, and player 2 received the next service in rally 4. Therefore, sequence 1 consists of the previous sideout 3 and next sideout 4. Sequence 4 aggregates the previous sideout in rally 3 and the next sideout in rally 10 by player 1. Rallies 8 and 9 do not interrupt sequence 4 because the length of the time window is three rallies only. One sideout can also be part of two sequences. For example, sideout in rally 6 is the next sideout in sequence 2 as well as the previous sideout in sequence 3. Sideouts in rallies 1 and 3 are not a sequence because they were played by different players. Sideouts in rallies 10 and 14 are also not a sequence, although they were played by the same player. The reason is that the time window between them is more than three rallies. The same applies to the pairs 1/7, 2/8, and 4/12.

For the purpose of this study, a *regular sideout* was defined as a sequence of three ball contacts (reception, set, and attack) directly after a serve. All other rallies, e.g., including service winners, attacks on the second ball contact or free balls, where a ball is passed over the net because an attack was not possible, were excluded. A *sideout sequence* consists of two regular sideouts by the same receiving/attacking player during the same set in a timeframe of four rallies, without being interrupted by a timeout. The first sideout in this sequence will be referred to as the *previous sideout* and the second sideout to as the *next sideout* (Figure 1).

For each sideout in a sideout sequence, the performance parameters *Technique* and *Outcome* were collected. Technique indicates whether the attacker hit the ball in a downward direction toward the floor (s*pike*) or softly directed the ball to an open area of the court (s*hot*). Outcome indicates whether the attacker scored a direct point or the opposing team was not able to touch the ball more than once (successful or *hit*). Otherwise, the sideout is designated as an *unsuccessful* or a *miss*. A miss does not imply that the sideout team did not win really, since there is still the option to defend the counterattack and to score. The variables *Technique Previous Sideout* and *Outcome Previous Sideout* refer to performance parameters in the previous sideout, *Technique Next Sideout* and *Outcome Next Sideout* to the next sideout.

All data were annotated by professional beach volleyball analysts by using custom-made observation software (Link, 2014). Observations based on self-made video recordings were taken by a single camera located behind the court. Data were a part of a more detailed dataset, which was used to prepare Germany’s national teams for their competitions. Cohen’s κ statistics showed substantial to perfect agreement between two observers based on a subset of 121 sequences (*κ* = 0.93 up to 1.0).

### Statistical Analysis

The analysis uses sideout sequences as statistical units. Data are presented as incidence rates (#) and hit rates (Hit) of sideouts (in %) by the factor *Gender* (men and women). First, the *incidence base rate* (#_base_) and the *hit base rate* (Hit_base_) of all sideouts in the categories of Technique are analyzed. #_base_ is calculated as the number of sideouts in a category divided by the number of all sideouts, and Hit_base_ as the number of hits divided by the number of sideouts in this category. Second, the influence of Outcome Previous Sideout on *technique alternation rate* (#_alter_) and *technique* alternation *hit rate* (Hit_alter_) is reported. For each category, #_alter_ is defined as the number of sequences, where Technique Previous Sideout and Technique Next Sideout differ, divided by the number of all sequences in this category. Hit_alter_ is calculated as the number of successful next sideouts, in which technique is alternated, divided by the number of all next sideouts of this kind. Third, the analysis reports effects of Technique Previous Sideout × Outcome Previous Sideout on the next sideouts played as shots. S*hot rate* (#_shot_) represents the number of next sideouts played as shots divided by the number of all next sideouts, and *shot hit rate* (Hit_shot_) the number of successful next sideouts played as shots divided by the number of next sideouts played as shots. In other words, #_shot_ indicates the share of next sideouts played as shots (and not as spikes), and Hit_shot_ describes how successful these shots are. For next sideouts played as spikes, *spike rate* (#_spike_) and *spike hit rate* (Hit_spike_) were calculated in a similar way.

When comparing two rates between conditions, their difference (Δ) (in ± %) of the reference value is reported. To test the significance of differences between rates, *χ*^2^-tests are conducted. Cramer’s *v* is used to describe the effect size of significant differences. Before using parametric statistical test procedures, the assumption of normality was verified. The α-level was set to 0.05. The Holm-Bonferroni method was applied to counteract the problem of multiple comparisons. All statistical analyses were performed using *R* (v3.5).

## Results

**Figure 2:**
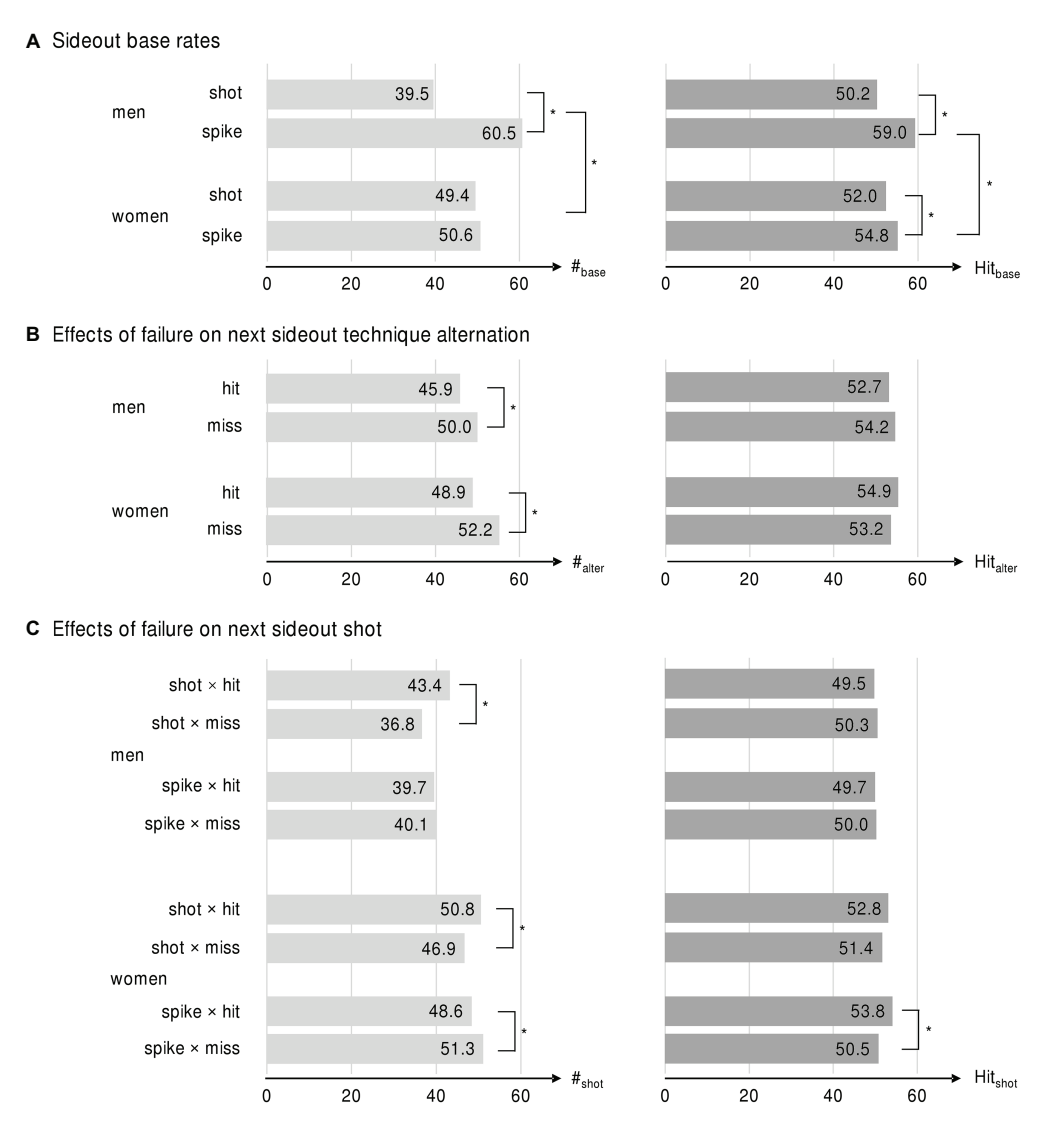
Part **(A)** shows base incidences rates (#_base_) and base hit rates (Hit_base_) of shots and spikes in sideouts grouped by Gender. Part **(B)** reports technique alternation rate (#_alter_) in next sideouts and hit rates (Hit_base_), in next sideouts, in which technique is alternated, grouped by Gender and Outcome Previous Sideout. Part **(C)** shows incidence rates of shots (#_shot_) and hit rates of shots (Hit_base_) in next sideouts grouped by Gender, Technique Previous Sideout and Outcome Previous Sideout. * indicates significant differences between conditions.

In the sample, there were 79,422 rallies in total, including 28,974 sideout sequences (12,755 for men and 16,219 for women). Analysis of incident base rates in the categories of Gender × Technique indicates more spikes than shots in men’s beach volleyball (Δ = +53.2%, $\chi^{2}$ = 272.3, *p* < 0.001, *v* = 0.10; Figure 2A). In women’s matches, no quantitative differences in the use of techniques were found. For men, the hit base rate of spikes was +17.5% higher than shots ($\chi^{2}$ = 98.8, *p* < 0.01, *v* = 0.09), and female players were +5.4% more successful when playing spikes ($\chi^{2}$ = 13.4, *p* < 0.01, *v* = 0.03). The hit base rate of spikes was +7.7% higher in men’s matches than women’s matches ($\chi^{2}$ = 27.8, *p* < 0.01, *v* = 0.05). No significant differences in hit rates of shots were found between men and women. In addition, no differences in base rates were found between the previous and next sideouts for either gender.

Analysis of performance in the categories of Gender × Outcome Previous Sideout shows a higher technique alternation rate in the next sideouts for male and female players after misses than the next sideouts after hits (men: Δ = +32.7%, $\chi^{2}$ = 61.0, *p* < 0.01, *v* = 0.07; women: Δ = +40.4%, $\chi^{2}$ = 117.1, *p* < 0.01, *v* = 0.08; Figure 2B). The technique alternation hit rate did not differ between hits and misses in the previous sideouts.

Significant Gender × Technique Previous Sideout × Outcome Previous Sideout interactions were found for sideouts played as shots (Figure 2C). After shot misses, shot rate in the next sideouts was −17.9% lower for men and −8.3% lower for women than the next sideouts after shot hits (men: $\chi^{2}$ = 22.1, *v* = 0.07, *p* < 0.01; women: $\chi^{2}$ = 11.9, *v* = 0.04, *p* < 0.01). After shot misses, no differences in shot hit rates were found for men and women when compared to sideouts after.

After spike misses, shot rate of female players in next sideouts was +5.5% higher ($\chi^{2}$ = 5.80, *v* = 0.03, *p* < 0.05) and shot hit rate was −6.5% lower than the next sideouts after spike hits ($\chi^{2}$ = 21.9, *v* = 0.07, *p* < 0.01). In men’s matches, there was no difference between these conditions. Analysis of the previous sideouts played as spikes shows no significant differences in spike rates and spike hit rates between any subgroup. Therefore, these results are not reported in Figure 2C.

## Discussion

### Discussion of Methods

The aim of the study was to answer the question whether sideout failure influences performance in the next sideouts. The definition of a miss includes not only direct errors like out or blocked balls but also defended attacks in which the ball was somehow controlled by the defending team (operationalized by two ball contacts). The idea behind this definition is that top-level teams will likely score when they are able to control the ball in defense (Giatsis et al., 2015), and therefore, the sideout player is expected to try to score with the first chance. Against this, one could argue that the psychological effect of direct errors might be higher than defended attacks because here scoring is still possible. The analysis is limited by the fact that it only studied outcome and technique of the next sideout. Failure could also affect other game actions such as service, block, reception, and setting or the direction of the attack.

By analyzing pairs of sideouts, the question is only whether failure in one sideout affected the next sideout. A different option would be to use streaks of more than one unsuccessful sideout (e.g., three, as proposed by Csapo and Raab, 2014). One argument for longer streaks might be that the effect of failure in beach volleyball is larger or even occurs after many misses in a row. On the other hand, this would lead to a much smaller number of sequences and decrease statistical power. The grounds for using a timeframe maximum of four rallies between two sideouts are that this produces the largest effects. Shorter timeframes exclude more sideouts in which the effect of failure was still present, whereas longer timeframes include more sideouts, which were not influenced by misses. Further studies could vary the timeframe and the streak length to explore the influence of these factors.

The statistical analysis of sequences uses probabilities between the conditions miss and hit (Raab et al., 2012). A different option would have been to treat attacks as continuous sequences and to analyze them by auto correlation and run test, as in the initial study by Gilovich et al. (1985) and many others. However, this approach would not be able to take proper account of confounding factors like timeouts, new sets, and longer timespans in which a player has no sideout. When calculating conditional probabilities, all players are treated as one group (e.g., Stern, 1995). Since this approach masks individual conditions, the results refer to the average performance of players only. Future studies could analyze the rate of players, which are affected by negative streaks and make statements on the range of effect sizes. For practical purposes in particular, it is also necessary to analyze players on an individual level (see paragraph “Discussion of Application”).

### Discussion of Results

The analysis of base rates suggests that the tactical decision for a particular technique was influenced by its success rate. Since spikes were more successful in men’s beach volleyball than shots, athletes might have used this technique more often, whether consciously or unconsciously. In women’s matches, the almost similar incidence base rates of spikes and shots are in line with their equal chance of success. The higher hit base rate of spikes in male competitions than women can be explained by motoric and anthropometric differences between genders (Thomas and French, 1985). This also implies that higher physical performance leads to bigger advantages for attacking than for defending actions.

The analysis of sideout pairs indicates that failure in a previous sideout leads to a general tendency to alternate attacking technique (question 1) in the next sideout for both men and women (Figure 2B). A lower hit rate after misses was found only in women’s beach volleyball (question 2). This is in line with Raab et al. (2012), who also reported a “cold-hand” effect in indoor volleyball but without differentiating between genders, technique, and sideout/defense situation. The effects only occur in particular constellations (question 3). Both, men and women, show a tendency not to play shot again when the previous shot was a miss (Figure 2C). We assume that athletes did not wanted to give the opponent a chance to run for a shot and therefore used a hard driven spike more often. This effect was higher in men’s beach volleyball than women, which is reasonable from a tactical perspective since hit rates in spike were higher in men’s matches (Figure 2A).

Women show a lower hit rate when playing a shot after they were not successful in playing a spike. One explanation could be that these shots were played less precise – either by playing less riskily to prevent a direct error or because of higher psychological pressure caused by failure in the previous sideout. A second cause could be that the defending teams anticipated the shots and consequently adapted their tactics – e.g., using a fake block or a different starting position in defense. In men’s beach volleyball, there was no effect of misses on hit rate of next sideouts maybe because shots play a less important role.

Effect sizes in the study are quite small. This is less surprising since performance streaks in general are infrequent, weak events are difficult to detect (Iso-Ahola and Dotson, 2014). Tactical decisions in complex sports like beach volleyball depend on a number of additional factors such as the quality of the players themselves and the pairs, situational awareness, tactical agreements, quality of setting, individual preference, climatic conditions, set time, the set number, or anthropometric factors, especially concerning the attacker and the blocker. Clearly, it is also the goal of every player to act as variably and unpredictably as possible. The elite athletes in the sample are at the level they are because they are able to put this into practice. Elite players must also have a high mental performance and stability, which reduces effects of failure. Nevertheless, sequence effects in beach volleyball are found, which support the existence of psychological momentum in this sport.

### Discussion of Application

**Figure 3:**
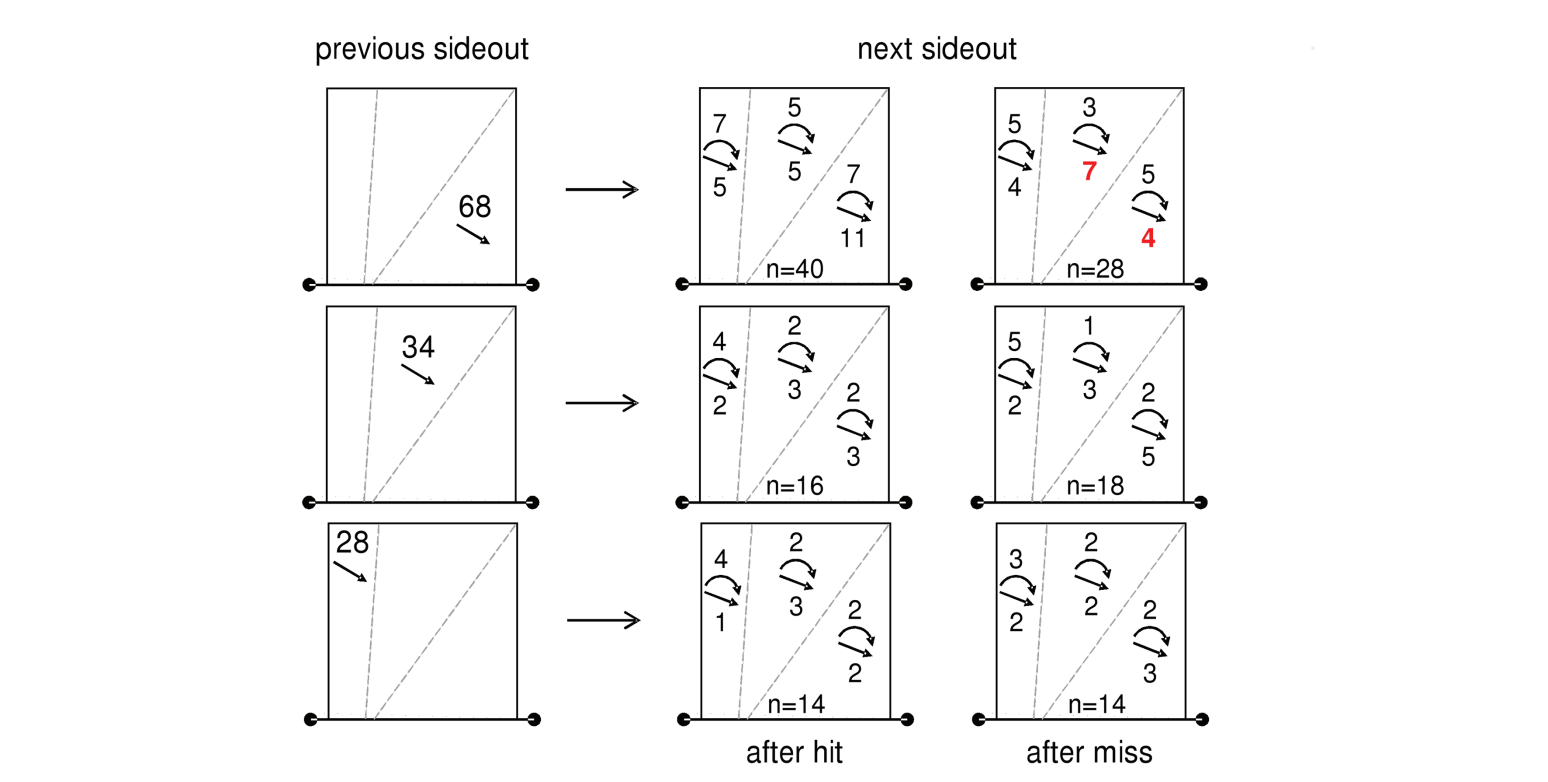
Match report used for supporting German national beach volleyball teams. It shows the profile of one female player in eight matches. Data include sideout sequences in which the previous sideout was played with a spike. The three courts in the left column show the number of spikes played diagonal (*n* = 68), mid (*n* = 34), and line (*n* = 28) in the previous sideouts. The other courts show direction (line, mid, diagonal) and technique (curved line with arrow ≙ shot, straight line with arrow ≙ spike) of attacks in the next sideouts after successful sideouts (second column) and after failure (third column). In this structure, e.g., the second court in the third row shows data of all sideouts after successful line spikes.

We think that the application of action sequences in practical game analysis needs additional performance variables, e.g., the attacking direction. Figure 3 shows a single case analysis similar to those that were used by the German teams during the 2012 and 2016 Olympics. The visualization includes data of sideout sequences of one top-level female player in eight matches in which the previous sideout was played with a spike. The three courts in the left column show the number of spikes played diagonal, mid, and line in the previous sideouts. The other courts show the number of smashes (straight line with arrow) and shots (curved line with arrow) in the next sideouts also grouped by their target zone. The courts in the second column show data of the next sideouts after hits, the courts in the third column after misses.

As far as we interpret the data, the player has a tendency to play two diagonal spikes in a row after she was successful with the first (11 diagonal spike in 39 sideouts, 28.2%). When she was not successful with the first diagonal spike, she repeated this technique only in 4 of 28 (14.3%) sideouts. Instead, she uses more spikes in the mid area of the court (7 mid spikes in 28 sideouts, 25.0%), compared to sideouts after hits (5 mid spikes in 39 sideouts, 12.8%). Consequently, teams who play against this player could try to adapt their defense, e.g., by trying to cover more of the central area of the court while blocking – without of course revealing this too early. After spikes played line or in the middle of the court (second and third row), the player shows no differences in attacking behavior between hits and misses.

It is easy to accept that these kinds of analytics can only provide first quantitative hints, which require confirmation using qualitative video analysis (Gréhaigne et al., 2001; Wilson, 2008). In addition, this information does not guarantee the correct prediction of individual behavior, since success in previous rallies is only one factor, which might affect performance. Validity depends on many considerations, e.g., if data were collected in balanced and high important matches in which the player was under pressure. In the final analysis, action sequences are only an additional information source for improving teams’ success probability. However, based on our experience, winning one or two points per match in top-level beach volleyball using this information makes the approach rewarding.

## Conclusion

The findings support the belief that attacking failure influences the choice of attacking technique in the next sideout in elite beach volleyball. In women’s beach volleyball failure has a negative impact on the hit rate in the next sideout, which provides supporting evidence for a cold-hand phenomena under certain conditions. In men’s beach volleyball players show a tendency to switch attacking technique after misses, but this does not influence the hit rate. Analysis of streaks on an individual level holds potential for practice, since the knowledge about behavioral stereotypes is a precondition for overcoming them by training and assisting a player in acting more unpredictably. They can also be used for developing match strategies, e.g., by better anticipating an opponent’s behavior after misses.

## Ethics Statement

Since each player agreed to the video recording of matches on signing their player license, an ethics approval was not required as per applicable institutional and national guidelines. Nevertheless, all procedures performed in the study were in strict accordance with the Declaration of Helsinki as well as with the ethical standards of the local ethics committee.

## Author Contributions

DL contributed to the conception, design of the study, and statistical analysis and wrote the manuscript. SW worked on data processing and data analysis. All authors contributed to manuscript revision, as well as reading and approving it for publication.

### Conflict of Interest Statement

The authors declare that the research was conducted in the absence of any commercial or financial relationships that could be construed as a potential conflict of interest.
